# The Effect of Digital Therapy–Based Aerobic Exercise on Children With Neuroblastoma: Protocol for a Prospective Controlled Trial

**DOI:** 10.2196/72698

**Published:** 2026-01-08

**Authors:** Xuan Zhou, Shihao Huang, Lixia Wang, Haibin Guo, Sisi Chen, Tingrui Li, Qing Du

**Affiliations:** 1 Department of Rehabilitation Medicine Xin Hua Hospital Shanghai Jiao Tong University School of Medicine Shanghai, Shanghai China; 2 Department of Pediatric Hematologic Oncology Xin Hua Hospital Shanghai Jiao Tong University School of Medicine Shanghai, Shanghai China

**Keywords:** rehabilitation, aerobic exercise, digital therapy, neuroblastoma, pediatrics

## Abstract

**Background:**

Neuroblastoma, one of the most common extracranial solid tumors in children, often results in significant functional impairments, including muscle atrophy, reduced motor function, and sensory disturbances. While aerobic exercise has been shown to support functional recovery in patients with cancer, digital therapy–based aerobic exercise interventions have not yet been explored in pediatric neuroblastoma.

**Objective:**

This study aims to develop and evaluate a digital intervention therapy to provide a safe, feasible, and tolerable home-based physical activity initiation protocol for children with neuroblastoma after chemoradiotherapy, with the main objectives of (1) breaking sedentary behavior habits and increasing daily energy expenditure, (2) gradually improving physical function (flexibility, basic muscle strength and muscle endurance, and balance ability), (3) establishing the habit of regular exercise and improving exercise self-efficacy, and (4) laying the foundation for a possible transition to more standard, albeit low-risk, aerobic exercise.

**Methods:**

A total of 68 participants will be recruited and allocated to 2 groups matched in age and gender, with consideration given to the preferences of participants and their guardians. The control group will receive standard in-hospital medical treatment and health education, whereas the experimental group will receive a 6-month digital therapy–based aerobic exercise intervention in addition to standard care. Outcomes will be assessed at baseline and 3 and 6 months, with data collected for analysis. The primary outcomes are the 6-minute walk distance and the timed scores (in seconds) for the timed up and go and stair climb tests. The secondary outcomes include body composition, grip strength, and quality of life measured using the Pediatric Quality of Life Inventory version 4.0.

**Results:**

Recruitment will be conducted from December 2025 to September 2026. We expect that the intervention group will show significantly greater improvements in physical function, physical activity levels, and fatigue management than the control group, demonstrating the effectiveness of digital therapy for rehabilitation training after radiotherapy and chemotherapy for neuroblastoma in children.

**Conclusions:**

We expect that the results of this study will provide evidence for rehabilitation after chemoradiotherapy in pediatric patients with neuroblastoma to improve physical function, physical activity levels, and fatigue management. In the future, expanding to multiple centers to conduct targeted surveys of different types of childhood cancers will help validate the effectiveness of digital therapy.

**International Registered Report Identifier (IRRID):**

DERR1-10.2196/72698

## Introduction

Neuroblastoma is one of the most common solid tumors in children, accounting for 8% to 10% of all childhood cancers, with a global incidence of approximately 10.1 cases per million children annually [1,2]. The clinical presentation of neuroblastoma varies depending on the tumor’s location and severity, often involving compression or invasion of neural structures. Standard treatments such as radiotherapy, chemotherapy, immunotherapy, and surgery can lead to adverse effects, including fatigue, vomiting, and malnutrition [3,4]. Additionally, children with neuroblastoma frequently experience limb motor dysfunction (eg, hypokinesia), sensory deficits, behavioral and cognitive impairments (eg, learning difficulties and attention-deficit/hyperactivity disorder), mood disorders, and sleep disturbances [5,6]. Given that childhood is a critical period for physical and mental development, the long-term impact of neuroblastoma on cardiopulmonary fitness and quality of life underscores the importance of early exercise interventions [7].

There is a growing body of preclinical evidence suggesting that exercise positively influences the tumor microenvironment, enhances immune function, and supports nervous system health [8,9]. In clinical settings, exercise has been shown to accelerate postoperative recovery, improve physical activity levels and cardiopulmonary fitness, and alleviate cancer-related fatigue in patients undergoing radiation or chemotherapy [10]. Combined exercise interventions alongside chemotherapy and surgery have been reported to reduce tumor growth rates by 31% to 67% and lower the risk of metastasis [11]. Furthermore, preoperative exercise interventions may decrease postoperative complications and shorten hospital stays [12]. Braam et al [13] demonstrated that exercise interventions during and after cancer treatment improve body composition, flexibility, cardiorespiratory fitness, muscle strength, and quality of life in pediatric patients with cancer. A systematic review of exercise interventions in children with acute lymphoblastic leukemia highlighted their benefits in reducing fatigue during chemotherapy and maintaining physical activity, muscle strength, bone density, and functional capacity after treatment [14].

Aerobic exercise, the most commonly studied form of exercise intervention in oncology, has been shown to enhance cardiovascular fitness, boost metabolism, strengthen muscles, improve quality of life, reduce fatigue, and alleviate depression [15-17]. For instance, Leclerc et al [18] reported significant improvements in motor function, body fat percentage, and quality of life in patients with breast cancer following a 3-month multidisciplinary aerobic exercise program. Similarly, Tanriverdi et al [19] found that a virtual reality–based aerobic exercise intervention improved sleep quality in children with acute lymphoblastic leukemia. Despite these benefits, patient adherence remains a significant barrier to the implementation of exercise interventions. Blanchard et al [20] noted that 52.7% to 70.4% of patients with cancer in the United States fail to meet recommended exercise guidelines. Strategies to enhance adherence and intervention efficacy are critical for advancing this field. For example, Le et al [21] demonstrated that a 12-week telephone-based exercise intervention improved physical activity, fatigue, quality of life, and vitality in survivors of cancer. However, such interventions require substantial manpower and lack direct interaction, highlighting the need for more scalable and interactive solutions.

The integration of digital health technologies into health care services has opened up new avenues for innovation. Digital therapeutics (DTx), which use evidence-based software programs to deliver interventions for disease treatment, management, or prevention, represent a promising approach [22]. DTx leverage artificial intelligence to enable effective disease monitoring, provide personalized treatment plans, and enhance health care delivery [23]. In 2019, the World Health Organization endorsed digital rehabilitation as a continuous care model in its guidelines on digital interventions for health system strengthening [24]. DTx for aerobic exercise interventions are now widely used in managing chronic conditions such as heart disease, diabetes, and obesity [25]. Studies have shown that users of digital platforms are more likely to achieve exercise-related benefits compared to nonusers [26], and DTx have demonstrated high engagement and acceptability among children and their parents [27]. Compared with traditional face-to-face aerobic training, DTx-based aerobic exercise offers several distinctive advantages, including real-time feedback, science popularization and patient education, and adaptive intensity adjustment through data analytics. These features contribute to higher user motivation, better adherence, and more precise monitoring of exercise quality and safety [28,29]. By transcending time and space limitations, DTx enable real-time remote guidance, intervention quality monitoring, and reduced hospitalization frequency, thereby improving intervention efficiency and alleviating the burden on health care providers.

While animal studies have demonstrated the positive effects of exercise on physical function, immunity, and tumor prognosis in neuroblastoma models [30], clinical evidence on the impact of DTx-based aerobic exercise in children with neuroblastoma remains scarce. This study aims to address this gap by investigating the effects of a novel aerobic exercise–based digital therapy intervention on motor function and quality of life in children with neuroblastoma.

## Methods

### Study Design

**Figure 1:**
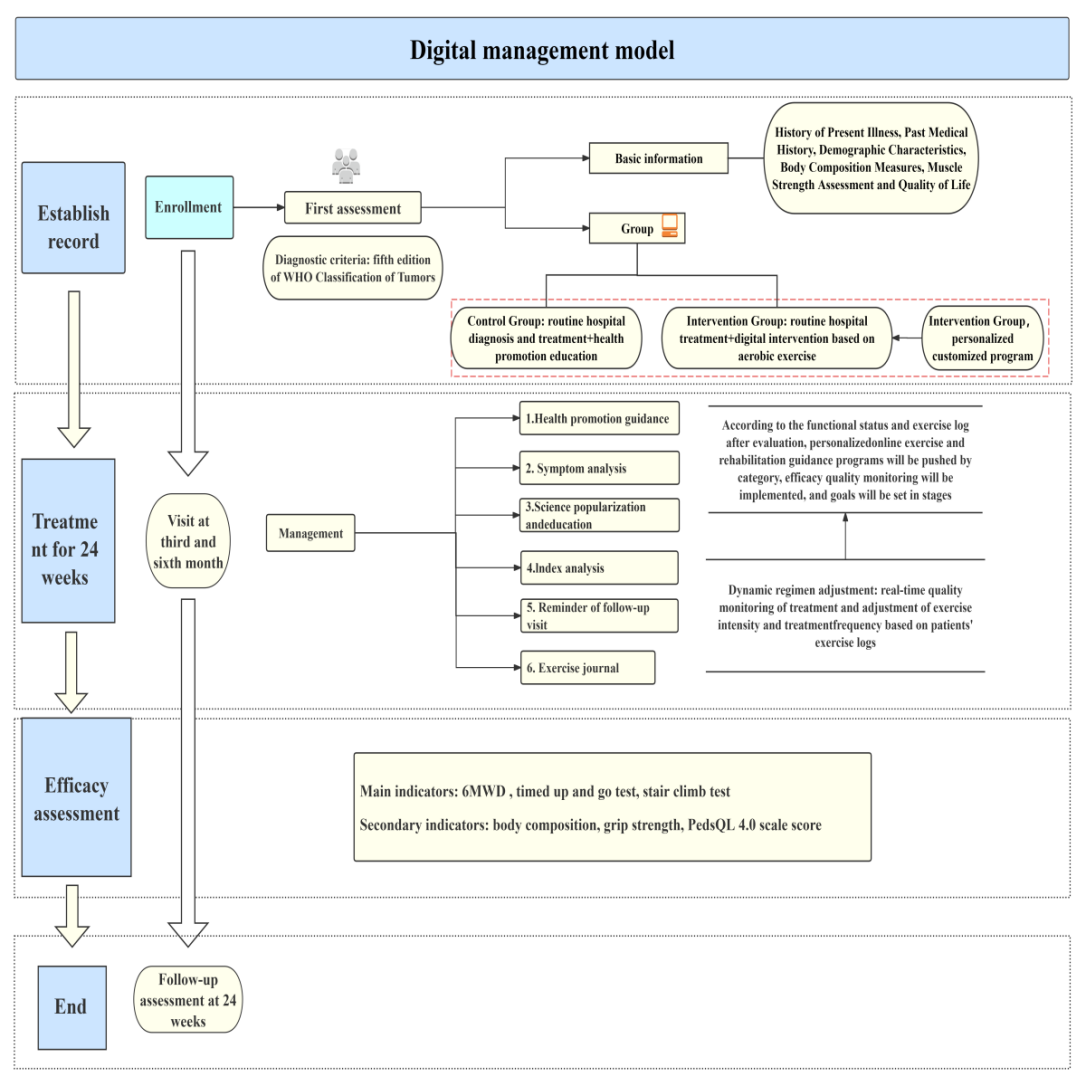
Digital management pathway for children with neuroblastoma. 6MWD: 6-minute walk distance; PedsQL 4.0: Pediatric Quality of Life Inventory version 4.0; WHO: World Health Organization.

This study is a single-center, prospective clinical trial designed to evaluate the efficacy of a digital therapy–based aerobic exercise intervention in children with neuroblastoma. The trial adheres to US Food and Drug Administration certification and management standards for digital therapy. Participants will be divided into 2 groups: the control group will receive routine in-hospital diagnosis and treatment, whereas the experimental group will receive a digital therapy–based aerobic exercise intervention in addition to standard care. Following group allocation, the control group will receive physical activity guidance from clinicians as part of conventional care, whereas the experimental group will follow a personalized digital exercise management program tailored to their disease condition and lifestyle (Figure 1). The trial aims to establish a digital management model for children with neuroblastoma through statistical analysis of clinical outcomes.

### Participants

Children aged 6 to 17 years with a confirmed diagnosis of neuroblastoma will be recruited from outpatient clinics at the research center. Participants will be recruited based on inclusion and exclusion criteria.

### Inclusion Criteria

The inclusion criteria are as follows: (1) confirmed diagnosis of neuroblastoma requiring radiotherapy or chemotherapy, (2) age of 6 to 17 years, (3) ability to understand and express themselves to some degree, (4) ability of the patient or their guardian to operate a smartphone app, (5) awareness of the patient’s condition and prognosis by the patient or their guardian, and (6) voluntary participation with signed informed consent from the patient and their guardian.

### Exclusion Criteria

The exclusion criteria are as follows: (1) preexisting malignant tumors or other types of solid or nonsolid tumors; (2) disease progression or recurrence at the time of study enrollment; (3) heart rate (HR) exceeding 70% of the age-specific maximum or below 60 beats per minute, systolic blood pressure of >180 mm Hg or orthostatic hypotension, respiratory rate of >50 breaths per minute, body temperature of >37.5 °C, or oxygen saturation of <90%; (4) occurrence within 3 months before enrollment of cardiac insufficiency (New York Heart Association functional class II or higher), severe pulmonary function decline, fracture, hypertension of grade ≥2, or concurrent use of ≥2 antihypertensive medications; (5) history of HIV infection, active hepatitis B virus or hepatitis C virus infection, or uncontrolled systemic infection; and (6) major surgery within 4 weeks before enrollment or planned major surgery during the study period.

### Grouping and Masking

Participants with a clear preference for either intervention will be allocated accordingly, whereas those without a preference will be randomly assigned to the experimental or control group. This approach was adopted to accommodate the preferences of the participants and their guardians while preserving the benefits of randomization. Due to the nature of the intervention, participants and intervention providers will not be blinded to group allocation. However, outcome assessors and statisticians will remain blinded to group assignments to minimize bias.

### Intervention

#### Experimental Intervention Program

The experimental group will undergo a baseline assessment using a self-developed questionnaire including demographic and disease information (obtained from hospital records), height, weight, BMI, 6-minute walk distance (6MWD), body composition, grip strength, and Pediatric Quality of Life Inventory version 4.0 (PedsQL 4.0) score. On the basis of the assessment results, a rehabilitation therapist will design a personalized aerobic exercise intervention program, including exercise prescriptions, home exercise routines, physical activity promotion, and daily activity management.

A self-developed digital management system will provide real-time monitoring of exercise logs and deliver tailored exercise strategies. The intensity of aerobic exercise will be adjusted based on individual fatigue levels measured using the Borg fatigue scale. The intervention will be conducted once daily for 30 minutes, 5 days per week over 24 weeks. The digital platform will also offer educational content and personalized information based on the patient’s condition and exercise progress.

#### Outline of the Exercise-Based Digital Therapy

The aerobic digital intervention exercise training program is tailored to participants’ Borg fatigue scale scores, with 3 intensity levels designed to accommodate varying levels of fatigue, as shown in Table 1. Each program requires 30 minutes of exercise per day, 5 days per week for 6 months.

**Table 1:** Exercises included in each program.

Exercises		Description
**Low-intensity program (Borg fatigue score 6-10)**		
	Seated arm stretch	Participants sit upright with their fingers crossed. They straighten their arms and raise their palms above their head. They hold for 30 seconds to feel a stretch in their arms and upper chest.
	Backward arm lift	Participants cross their fingers behind their back with their palms facing each other. They slowly turn their elbows inward (palms outward) while straightening their arms. They lift their arms upward and away from their hips until they feel a stretch in their arms, shoulders, or chest. They hold for 30 seconds, keeping their chest lifted and chin tucked.
	Seated leg stretch	Participants sit on the edge of a chair with one leg extended and the heel on the floor. They place their hand on the opposite thigh for support. They bend slightly forward at the hips until they feel a stretch in the back of the extended leg. They hold for 10 to 15 seconds and then switch legs.
	Supported knee bend	Participants face a wall or stable surface for support. They place their forearm on the wall and rest their head on the back of their hand. They bend one knee and bring it close to the support, keeping the other leg straight. They hold for 30 seconds and then switch legs.
	Heel-to-buttock stretch	Participants hold the top of their right foot with their left hand and gently pull their heel toward their buttocks. They hold for 30 seconds and then switch legs.
	Waist stretch	Participants stand with their feet shoulder-width apart. They cross their left hand over their waist and raise their right hand above their head. They bend their upper body to the left, hold for 15 seconds, and repeat on the opposite side.
**Moderate-intensity program (Borg fatigue score 11-12)**		
	Standing heel lift	Participants stand upright with their feet shoulder-width apart and arms hanging naturally. They lift both heels off the ground, extending their feet fully. They hold for 5 seconds, then lower their heels and repeat 5 times.
	Lunge with arm raise	Participants stand with their feet shoulder-width apart and hands on their hips. They lunge forward with their left leg, bending both knees until their right knee nearly touches the ground. They raise their arms above their head, interlocking their palms. They hold for 15 seconds and then return to the starting position.
	One-legged stand	Participants stand near a wall for support. They lift one foot slightly off the ground, bending the other knee. They hold for up to 30 seconds and then switch legs.
	Back stretch	Participants position themselves on all fours with their hands under their shoulders and their knees under their hips. They arch their back upward, holding for 10 seconds, and then lower their back slightly. They repeat for 30 to 60 seconds.
	Standing shrug	Participants stand with their feet shoulder-width apart, chest up, and core engaged. They let their arms hang naturally at their sides with palms facing in. To perform the movement, they elevate their shoulders toward their ears in a controlled motion, hold the contraction briefly at the top, then slowly lower back to the start.
	Kneeling push-ups	Participants place their hands on the ground slightly wider than shoulder-width apart, with their knees hip-width apart. They bend their elbows to lower their chest toward the ground and then push back up.
**High-intensity program (Borg fatigue score 13-14)**		
	In-place high knee raises	Participants stand with their hands on their waist and upper body straight. They lift their right knee to form a right angle with their thigh and calf and then alternate with their left knee in a running motion.
	Deep squat	Participants stand with their back against a wall and feet slightly apart. They slide down the wall until their thighs are parallel to the floor, keeping their hips higher than their knees. They hold for 5 to 10 seconds and then return to the starting position.
	Knee straightening	Participants sit in a chair with their feet together. They straighten one knee, hold for a few seconds, and then slowly lower it and repeat with the other leg.
	Wall push	Participants place their hands at shoulder height on a wall with their fingers pointing upward. They bend their elbows to bring their body closer to the wall and then push back until their arms are straight.
	Plank support	Participants position themselves on their elbows and toes, keeping their body in a straight line from the shoulders to the ankles, and hold for 30 seconds.
	Step-up squat	Participants stand in front of a sturdy bench with one leg bent back and their toes on the bench. Participants bend their forward leg until their thigh is parallel to the floor and then return to the starting position. Participants complete 10 repetitions and then switch legs.

### Control Group Program

Children with neuroblastoma who meet the inclusion criteria will be introduced to the study’s purpose. After obtaining informed consent, a baseline survey will be conducted using a self-developed questionnaire that includes demographic and disease information extracted from the hospital’s information system. Additional baseline measurements include height, weight, BMI, 6MWD, body composition, grip strength, and PedsQL 4.0 score. The control group will receive standard in-hospital medical treatment and health education. At the 3- and 6-month follow-up visits, outcome assessments and questionnaires are administered to the control group (demographic and baseline disease information are not collected).

### Compliance Supervision

All personnel involved in the study will be qualified and have undergone prestudy training to ensure role clarity and standardization. Data collectors, data entry personnel, rehabilitation therapists, and digital therapy supervisors will complete training and pass examinations to ensure consistency. Participants in the experimental group will be added to a digital management platform (WeChat app) where they will log daily exercise sessions and receive educational content and virtual or physical rewards based on their adherence. A home training diary will be maintained by patients or their guardians on the digital platform to record exercise time and frequency. Quality control personnel will regularly monitor adherence through the platform. Participants who complete the study will receive additional small toy incentives or follow-up program instruction sessions. Adherence to digital therapy will be measured as the percentage of completed daily log-ins relative to the minimum required (80% adherence threshold). Participants with adherence below 80% will be contacted by the researchers to document reasons for noncompliance.

### Withdrawal Criteria

Participants will be withdrawn from the study for the following reasons: (1) serious violation of the test protocol by the participant or their guardian or (2) receipt of other treatments by the participants during the trial that interfere with the study.

### Suspension Criteria

This study will be suspended or terminated for participants under the following circumstances: (1) failure to complete the 6-month intervention as required by the study protocol, (2) occurrence of serious safety issues that compromise participant safety, (3) significant protocol deviations that hinder efficacy or safety evaluation, (4) a decision by the ethics committee to suspend or discontinue the study, or (5) a decision by the study sponsor to suspend or halt the study.

### Adverse Events

All adverse events (AEs) occurring during the trial will be documented in an AE form. Investigators will assess the relationship between AEs and the intervention and determine whether to discontinue the intervention. AEs will be followed up on until resolution.

### Comprehensive Assessment

Efficacy assessments will be conducted by qualified rehabilitation therapists at baseline and 3 and 6 months. Assessments will include (1) demographic and physical developmental indicators (height, weight, and BMI), (2) 6MWD, (3) timed up and go (TUG) test, (4) stair climb test (SCT), (5) body composition, (6) grip strength, and (7) PedsQL 4.0 score (Table 2).

**Table 2:** Study timeline.

			Screening entry period: visit 0 (day 1)		Treatment period: visit 1, week 12 (–6 days to +6 days)		Treatment period: visit 2, week 24 (–6 days to +6 days)
Informed consent			✓				
Inclusion and exclusion criteria			✓				
Basics			✓				
Measurement of human body parameters			✓		✓		✓
**Instrument-based test**							
	Body composition determination	✓		✓		✓	
	Muscle power	✓		✓		✓	
**Quality of life**							
	Score on the Chinese version of the PedsQL 4.0^a^	✓		✓		✓	
**Outcome measures**							
	6-minute walk to test walking distance	✓		✓		✓	
	Timed up and go test	✓		✓		✓	
	Stair climb test	✓		✓		✓	
	Adverse event assessment	✓		✓		✓	

^a^PedsQL 4.0: Pediatric Quality of Life Inventory version 4.0.

### Primary Outcome Measure

#### 6MWD Measure

The 6-minute walk test is conducted in a flat, 30-m corridor. Participants are instructed to walk as far as possible within 6 minutes without running or jogging. The total distance walked back and forth along the corridor during the 6-minute period is recorded. The distance is measured in meters with an accuracy of 0.1 m. Vital signs, including HR (pretest resting HR and immediate posttest HR), respiratory rate, blood pressure, oxygen saturation, and the Borg subjective fatigue score, are assessed and documented both before and after the test.

For the test environment, a 30 m–long corridor with marked lines at 1-m intervals will be used, and bright-colored strips are attached to the ground to indicate the start and end point of each 60-m segment.

The test requires that participants wear comfortable, walkable shoes; avoid strenuous exercise for at least 2 hours before the test; and rest for a minimum of 10 minutes before the test begins.

If a participant experiences physical discomfort during the test, they may take a short break by leaning against the wall and resume walking once they feel better. The timer will not be paused during this process. If the test is terminated early (before 6 minutes), the following information must be recorded: (1) the time at which the test was stopped, (2) the reason for early termination, and (3) the total distance walked up to that point.

#### TUG Test

The TUG test will be used to assess functional mobility due to its reliability, cost-effectiveness, and efficiency [31]. During the test, the participants will be instructed to rise from a chair, walk a distance of 3 m, turn around, return to the chair, and sit down again. The total time taken to complete this task will be quantified in seconds. Performance is inversely related to the score, meaning that a shorter time indicates better functional mobility.

#### SCT Measurement

The SCT will be used to assess functional mobility, including domains such as strength, speed, and axial movement (rotation). The SCT requires participants to ascend a 10-step staircase, promptly turn around at the top, and descend back to the starting point. Children will be instructed to perform the task as quickly and safely as possible without pausing and are permitted to use the handrails. The total completion time, recorded in seconds, serves as the primary outcome measure, with shorter times reflecting better performance [31].

### Secondary Outcome Measures

#### Body Composition Index

A body composition analyzer (InBody 260) will be used to measure the patients’ body composition: body fat percentage, basal metabolic rate, skeletal muscle mass, waist-to-hip ratio, body water, protein, and inorganic salt content.

#### Grip Strength

Grip strength will be measured using a TKK-5401 grip strength meter produced by Takei. The participants’ body will be in an upright position, with their feet shoulder-width apart, arms naturally hanging down, and palms inward and one hand holding the grip strength meter at full grip strength. During the measurement, the assessor must ensure that the grip strength meter does not touch the participant’s body or clothing. The value displayed on the device is then recorded by the assessor. The test is performed twice (alternating hands), a 15-second break is allowed between measurements, and the best results are retained for the stronger hand, with grip strength measured in kilograms accurate to 0.1 kg.

#### Score on the PedsQL 4.0 Scale

The Chinese version of the PedsQL 4.0 scale will be used in this study. This scale has demonstrated good reliability and validity and has been widely adopted in research assessing quality of life among children in China. The PedsQL 4.0 scale comprises 23 items, which are categorized into 4 domains: physical functioning, emotional functioning, social functioning, and school functioning. Each item is rated on a 5-point Likert scale ranging from 0 to 4. During scoring, responses are transformed into a scale from 0 to 100, where higher scores indicate better outcomes. The domain score is calculated by summing the scores of all items within that domain and dividing by the number of items in the domain. Similarly, the total summary score is derived by summing the scores of all 23 items and dividing by the total number of items. Both the domain scores and the total summary score range from 0 to 100, with higher scores reflecting a better quality of life.

### Statistical Analysis

Data will be analyzed using SPSS (version 26.0; IBM Corp). Primary models using the intention-to-treat principle will be conducted for all primary and secondary outcomes. Data normality will be assessed initially. Nonnormally distributed data will be presented as medians with IQRs. Between-group comparisons (intervention vs control) for these variables will be conducted using the Mann-Whitney *U* test. Within-group comparisons (baseline vs each follow-up) will be conducted using the Wilcoxon signed rank test. Categorical data will be reported as numbers with percentages and analyzed using the chi-square test. Differences in primary and secondary outcomes between the intervention and control groups across all assessment time points will be evaluated using mixed-model analysis. Statistical significance will be defined as a *P* value of <.05, with multiple comparisons adjusted using the Bonferroni method.

### Sample Size Calculation

The sample size was calculated using G*Power (version 3.1.9.7; Heinrich Heine University Düsseldorf). On the basis of a related study [32], an effect size of 0.953 was determined, derived from the difference in the 6MWD between the experimental group (mean 35.89, SD 8.46 laps) and the control group (mean 26.76, SD 10.57 laps) after the intervention. The significance level (α) was set at .05, and the power (1 – β) was set at 0.95. The calculation indicated that 30 participants were required for each group. To account for a potential 10% loss to follow-up, 34 participants per group were planned for recruitment, resulting in a total sample size of 68 participants.

### Quality Control and Quality Assurance

All researchers involved in this study possess appropriate clinical research qualifications. Before the commencement of the clinical trial, the project leader or principal investigator conducted comprehensive training sessions for the research team. Personnel responsible for evaluations were specifically trained in evaluation procedures and operational methods, with their competency regularly assessed. Quality control measures include monthly monitoring and evaluation by inspectors within each group, as well as quarterly monitoring and evaluation by the study team’s inspectors. To ensure the integrity of the study, a data and safety monitoring committee was established to oversee the quality of each process. The clinical research unit is responsible for maintaining the quality and safety of the study, ensuring both the safety of participants and the validity of the data. The trial may be terminated if the intervention is found to be ineffective or if significant safety concerns arise based on the assessment of data quality, efficacy outcomes, and AEs. In the event of an AE, the principal investigator will evaluate the situation and determine whether to suspend the intervention.

### Ethical Considerations

#### Ethics and Informed Consent

This study has been approved by the Medical Ethics Committee of Xinhua Hospital, affiliated to Shanghai Jiao Tong University School of Medicine (XHEC-C-2023-062), and all study participants will provide written informed consent.

#### Dissemination and Publication Policy

Study results will be published in peer-reviewed journals and presented at conferences and relevant stakeholder engagement activities. There will be no information that can identify participants in any publication. Participants who explicitly express a wish to be informed about the research outcome will be contacted and offered to receive an article or poster with a lay summary of the study.

## Results

Recruitment will be conducted from December 2025 to September 2026. It is expected that the intervention group will show significantly greater improvements in physical function, physical activity levels, and fatigue management compared to the control group and that the effectiveness of digital therapy for rehabilitation training after radiotherapy and chemotherapy for neuroblastoma in children can be demonstrated. The findings of this study are anticipated to be published by late 2026 or early 2027.

## Discussion

### Expected Findings

DTx represent an innovative treatment modality that facilitates real-time remote guidance and intervention quality monitoring. This approach reduces the need for frequent hospital visits while enhancing the efficiency of interventions [29]. Aerobic exercise has been widely recognized for its significant role during and after oncology treatment, contributing to improvements in physical fitness, muscle strength, and functional capacity. Additionally, it alleviates symptoms such as pain and diarrhea, shortens hospitalization duration, and promotes mental health and emotional well-being [33]. It should be pointed out that the primary goal of this “aerobic digital intervention” program is not to directly maximize cardiorespiratory endurance (such as maximal oxygen consumption) in the traditional sense but to provide a safe, feasible, and tolerable home physical activity initiation program for participants with different levels of fatigue to improve physical function, physical activity level, and fatigue management.

The integration of aerobic exercise interventions with DTx has shown promise in enhancing intervention efficacy through personalized, multilevel management of exercise programs. To the best of the authors’ knowledge, this study is the first controlled trial to evaluate the impact of an aerobic exercise intervention combined with digital therapy on motor performance in children with neuroblastoma.

The primary objective of this study is to establish a digital management intervention protocol for children with neuroblastoma by comparing the effectiveness of DTx combined with an aerobic exercise intervention against traditional in-hospital visits and health promotion strategies. The study outcomes are expected to demonstrate significant improvements in cardiopulmonary fitness, muscle strength, body composition, and quality of life. These findings will provide an evidence-based rationale for the future implementation of digital therapy–supported aerobic exercise programs in this population.

### Limitations

Despite its potential, this study has several limitations that should be considered when interpreting the results. First, the single-center design and small sample size restrict the generalizability of the findings. Future research should involve multiple centers across different regions and include larger samples to enhance external validity. Second, although outcome assessors and statisticians were blinded, participants and care providers were not. Furthermore, for participants with a preexisting preference, allocation was based on that preference rather than randomization. This nonrandomized allocation for a subset of participants introduces potential performance and expectation biases. As a preliminary investigation, this study provides foundational insights that can inform the design of a more rigorous randomized controlled trial in the future. Finally, the digital therapy platform and exercise programs are self-developed, and no formal large-scale usability testing was conducted before the intervention. While the design was informed by pediatric expertise and in-study engagement metrics supported feasibility, future iterations would benefit from structured usability testing to optimize the platform before broader implementation.

### Conclusions

This study represents an important first step in aerobic digital therapy for pediatric neuroblastoma. It provides evidence that rehabilitation after chemoradiotherapy in pediatric patients with neuroblastoma can improve physical function, physical activity levels, and fatigue management. In the future, expanding to multiple centers to conduct targeted surveys of different types of childhood cancers will help validate the effectiveness of digital therapy–based aerobic exercise.

## Abbreviations

6MWD: 6-minute walk distance
AE: adverse event
DTx: digital therapeutics
HR: heart rate
PedsQL 4.0: Pediatric Quality of Life Inventory version 4.0
SCT: stair climb test
TUG: timed up and go

## References

[1] Wahba A, Wolters R, Foster JH (2023). Neuroblastoma in the era of precision medicine: a clinical review. Cancers (Basel).

[2] 2Anderson J, Majzner RG, Sondel PM (2022). Immunotherapy of neuroblastoma: facts and hopes. Clin Cancer Res.

[3] Schirrmacher V (2019). From chemotherapy to biological therapy: a review of novel concepts to reduce the side effects of systemic cancer treatment (Review). Int J Oncol.

[4] Qiu B, Matthay KK (2022). Advancing therapy for neuroblastoma. Nat Rev Clin Oncol.

[5] Croteau N, Nuchtern J, LaQuaglia MP (2021). Management of neuroblastoma in pediatric patients. Surg Oncol Clin N Am.

[6] Zheng DJ, Krull KR, Chen Y, Diller L, Yasui Y, Leisenring W, Brouwers P, Howell R, Lai J, Balsamo L, Oeffinger KC, Robison LL, Armstrong GT, Kadan-Lottick NS (2018). Long-term psychological and educational outcomes for survivors of neuroblastoma: a report from the Childhood Cancer Survivor Study. Cancer.

[7] Butler E, Ludwig K, Pacenta HL, Klesse LJ, Watt TC, Laetsch TW (2021). Recent progress in the treatment of cancer in children. CA Cancer J Clin.

[8] Maddocks M (2020). Physical activity and exercise training in cancer patients. Clin Nutr ESPEN.

[9] Horowitz AM, Fan X, Bieri G, Smith LK, Sanchez-Diaz CI, Schroer AB, Gontier G, Casaletto KB, Kramer JH, Williams KE, Villeda SA (2020). Blood factors transfer beneficial effects of exercise on neurogenesis and cognition to the aged brain. Science.

[10] Van Dijk-Lokkart EM, Steur LM, Braam KI, Veening MA, Huisman J, Takken T, Bierings M, Merks JH, Van den Heuvel-Eibrink MM, Kaspers GJ, Van Dulmen-den Broeder E, Van Litsenburg RR (2019). Longitudinal development of cancer-related fatigue and physical activity in childhood cancer patients. Pediatr Blood Cancer.

[11] Buss LA, Dachs GU (2020). Effects of exercise on the tumour microenvironment. Adv Exp Med Biol.

[12] Hojman P, Gehl J, Christensen JF, Pedersen BK (2018). Molecular mechanisms linking exercise to cancer prevention and treatment. Cell Metab.

[13] Braam KI, van der Torre P, Takken T, Veening M, van Dulmen-den Broeder E, Kaspers GJ (2013). Physical exercise training interventions for children and young adults during and after treatment for childhood cancer. Cochrane Database Syst Rev.

[14] Coombs A, Schilperoort H, Sargent B (2020). The effect of exercise and motor interventions on physical activity and motor outcomes during and after medical intervention for children and adolescents with acute lymphoblastic leukemia: a systematic review. Crit Rev Oncol Hematol.

[15] Santos LS, Rehder MS, Negrao MV, Goes-Santos BR, Toshi Dias E, Paixão CJ, Urias U, Giannetti NS, Hajjar LA, Filho RK, Negrão CE (2024). Aerobic exercise training combined with local strength exercise restores muscle blood flow and maximal aerobic capacity in long-term Hodgkin lymphoma survivors. Am J Physiol Heart Circ Physiol.

[16] Campbell KL, Winters-Stone KM, Wiskemann J, May AM, Schwartz AL, Courneya KS, Zucker DS, Matthews CE, Ligibel JA, Gerber LH, Morris GS, Patel AV, Hue TF, Perna FM, Schmitz KH (2019). Exercise guidelines for cancer survivors: consensus statement from international multidisciplinary roundtable. Med Sci Sports Exerc.

[17] Rodríguez-Cañamero S, Cobo-Cuenca AI, Carmona-Torres JM, Pozuelo-Carrascosa DP, Santacruz-Salas E, Rabanales-Sotos JA, Cuesta-Mateos T, Laredo-Aguilera JA (2022). Impact of physical exercise in advanced-stage cancer patients: systematic review and meta-analysis. Cancer Med.

[18] Leclerc AF, Foidart-Dessalle M, Tomasella M, Coucke P, Devos M, Bruyère O, Bury T, Deflandre D, Jerusalem G, Lifrange E, Kaux J, Crielaard J, Maquet D (2017). Multidisciplinary rehabilitation program after breast cancer: benefits on physical function, anthropometry and quality of life. Eur J Phys Rehabil Med.

[19] Tanriverdi M, Cakir E, Akkoyunlu ME, Cakir FB (2022). Effect of virtual reality-based exercise intervention on sleep quality in children with acute lymphoblastic leukemia and healthy siblings: a randomized controlled trial. Palliat Support Care.

[20] Blanchard CM, Courneya KS, Stein K, American Cancer Society's SCS-II (2008). Cancer survivors' adherence to lifestyle behavior recommendations and associations with health-related quality of life: results from the American Cancer Society's SCS-II. J Clin Oncol.

[21] Le A, Mitchell HR, Zheng DJ, Rotatori J, Fahey JT, Ness KK, Kadan-Lottick NS (2017). A home-based physical activity intervention using activity trackers in survivors of childhood cancer: a pilot study. Pediatr Blood Cancer.

[22] Hong JS, Wasden C, Han DH (2021). Introduction of digital therapeutics. Comput Methods Programs Biomed.

[23] Dang A, Arora D, Rane P (2020). Role of digital therapeutics and the changing future of healthcare. J Family Med Prim Care.

[24] (2019). Recommendations on digital interventions for health system strengthening. World Health Organization.

[25] Moschonis G, Siopis G, Jung J, Eweka E, Willems R, Kwasnicka D, Asare BY, Kodithuwakku V, Verhaeghe N, Vedanthan R, Annemans L, Oldenburg B, Manios Y (2023). Effectiveness, reach, uptake, and feasibility of digital health interventions for adults with type 2 diabetes: a systematic review and meta-analysis of randomised controlled trials. Lancet Digit Health.

[26] Parker K, Uddin R, Ridgers ND, Brown H, Veitch J, Salmon J, Timperio A, Sahlqvist S, Cassar S, Toffoletti K, Maddison R, Arundell L (2021). The use of digital platforms for adults' and adolescents' physical activity during the COVID-19 pandemic (our life at home): survey study. J Med Internet Res.

[27] Eppes EV, Augustyn M, Gross SM, Vernon P, Caulfield LE, Paige DM (2023). engagement with and acceptability of digital media platforms for use in improving health behaviors among vulnerable families: systematic review. J Med Internet Res.

[28] Li G, Zhou X, Deng J, Wang J, Ai P, Zeng J, Ma X, Liao H (2025). Digital therapeutics-based cardio-oncology rehabilitation for lung cancer survivors: randomized controlled trial. JMIR Mhealth Uhealth.

[29] Zhang ZY, Tian L, He K, Xu L, Wang XQ, Huang L, Yi J, Liu ZL (2022). Digital rehabilitation programs improve therapeutic exercise adherence for patients with musculoskeletal conditions: a systematic review with meta-analysis. J Orthop Sports Phys Ther.

[30] Rincón-Castanedo C, Martín-Ruiz A, Zazo S, Luis Huertas AL, Valenzuela PL, Morán M, Fleck SJ, Santos-Lozano A, Ramírez M, Rojo F, Lucia A, González-Murillo Á, Fiuza-Luces C (2023). Combined exercise intervention in a mouse model of high-risk neuroblastoma: effects on physical, immune, tumor and clinical outcomes. Exerc Immunol Rev.

[31] Sinanoglu B, Ozdemir F (2023). Evaluation of functional mobility, balance, and executive functions in children with epilepsy. Epilepsy Behav.

[32] Tanir MK, Kuguoglu S (2013). Impact of exercise on lower activity levels in children with acute lymphoblastic leukemia: a randomized controlled trial from Turkey. Rehabil Nurs.

[33] Knols R, Aaronson NK, Uebelhart D, Fransen J, Aufdemkampe G (2005). Physical exercise in cancer patients during and after medical treatment: a systematic review of randomized and controlled clinical trials. J Clin Oncol.

